# Design and evaluation of a blended basketball training program using the ADDIE model

**DOI:** 10.1371/journal.pone.0332820

**Published:** 2025-09-29

**Authors:** Jun Sun, Nipaporn Khamcharoen

**Affiliations:** 1 Faculty of Education, Dhonburi Rajabhat University (DRU), Bangkok, Thailand; 2 Department of Public Foundation, Wannan Medical College, Wuhu, China; Haliç University, Faculty of Science, TÜRKIYE

## Abstract

This study aimed to develop and evaluate a blended learning basketball training program for university students based on the ADDIE instructional design model. A quasi-experimental one-group pre-test–post-test design was conducted with 30 first-year undergraduates in Wuhu, China. Over five days (30 hours), participants engaged in online theoretical learning and offline practical sessions covering five basketball skill areas, followed by a satisfaction survey. All measured skills improved significantly (**p* *< .001), with large effect sizes in dribbling (*d* = 2.14) and passing (*d* = 3.34), while improvements in shooting and three-step layups were relatively smaller but still significant. Mean satisfaction was 4.07/5, with high ratings for instructional support and learning resources. These findings support the effectiveness of the ADDIE-based blended learning model in enhancing basketball skills and student engagement. This structured approach offers practical value for improving skill acquisition in sports education.

## 1. Introduction

The COVID-19 pandemic, which emerged at the end of 2019, severely disrupted normal teaching activities worldwide [1]. In response, universities swiftly transitioned to online instruction to ensure that students’ learning continued uninterrupted [2]. Physical education, inherently practical and interactive, has traditionally relied on face-to-face teaching methods. However, blended learning rapidly became a flexible and effective instructional strategy in the pandemic. This model not only adapts well to addressing public health crises but also facilitates the equitable distribution of educational resources [3]. As a result, blended learning has become an integral part of modern education systems [4,5], offering significant potential in physical education.

Research indicates that blended learning enhances flexibility, diversifies resources, and improves skill training outcomes [6]. Supported by online platforms, it enriches theoretical and demonstration resources, while overcoming time and space limitations of face-to-face teaching. Moreover, blended learning has been shown to improve students’ self-directed learning, satisfaction, motivation, and self-efficacy [7–9]. For example, one study reported 14.8% higher learning effectiveness and 14.4% higher satisfaction compared with online-only instruction [10], while another found more than 200% improvement in fundamental basketball skills such as passing and shooting accuracy [11]. Similar results have been observed across other physical education contexts, showing consistent gains in skill acquisition, motivation, physical fitness, and satisfaction [12,13].

Despite these advantages, challenges remain in applying blended learning to physical education. These include the stability and user-friendliness of technology platforms [14], students’ insufficient self-management skills [15], weak integration of online and offline components that limit teamwork [16], and the increased burden on teachers for preparation and motivation [17,18]. Recent studies suggest that integrating blended learning with instructional design frameworks can enhance instructional coherence, active learning, and skill performance [19]. For example, applying the TPACK framework in football education improved both technical skills and motivational outcomes [20], while the ADDIE model in college aerobics courses improved participation, skill acquisition, and teaching quality [21]. However, most existing studies have focused primarily on learning outcomes, with insufficient attention to the systematic aspects of instructional design [22,23]. This has led to a partial disconnection between students’ learning needs and instructional practices, underscoring the necessity of adopting more structured design approaches.

Among the various instructional design models, the ADDIE model stands out for its clear logic, comprehensive process, and strong applicability. Research has demonstrated its effectiveness in clarifying instructional objectives, optimizing resource alignment, and improving learning performance, particularly in skill-based and online learning environments [24,25]. Compared to the SAM model and the Dick and Carey model, the structured and sequential characteristics of ADDIE make it especially suitable for developing systematic and outcome-oriented curricula in blended physical education settings [26].

In response to this gap, the present study aims to design and evaluate a structured blended basketball training program for university students by fully integrating the ADDIE instructional design model. The ADDIE model (comprising five phases: Analysis, Design, Development, Implementation, and Evaluation) is widely recognized for its logical structure, goal-oriented planning, and iterative feedback mechanisms [27]. Each phase serves a specific function: identifying learner needs (Analysis), defining learning objectives and course content (Design), developing instructional materials (Development), delivering instructional activities (Implementation), and assessing outcomes to inform continuous improvement (Evaluation). By detailing each ADDIE phase and linking needs analysis to module design, materials, implementation, and evaluation artifacts, this study provides a transparent and replicable PE design workflow that is often missing in prior blended learning research.

Building on this rationale, the research hypothesizes that a blended basketball training course designed using the ADDIE model **i**s expected to significantly improve students’ fundamental basketball skills and increase their learning satisfaction. To evaluate this hypothesis, a blended training program was developed and implemented for university students in accordance with the ADDIE model. The program’s results were measured using pre- and post-intervention tests and a satisfaction survey. This study provides a practical framework for the design of blended learning in college physical education. It also contributes empirical support to instructional design theory in sports contexts and offers valuable insights for enhancing university-level physical education in the post-pandemic era.

## 2. Materials and methods

**Fig 1:**
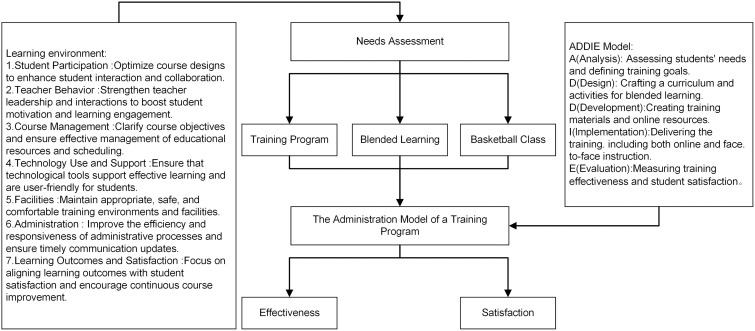
Administration model of a blended basketball training program based on the ADDIE framework.

This study employed a quantitative research design. Specifically, a cross-sectional descriptive survey was conducted to assess students’ needs and satisfaction, and a quasi-experimental pre-test–post-test design without a control group was used to evaluate the pilot implementation of the training program. The overall research framework was presented in Fig 1, which illustrated the development and evaluation process of the training program, integrating students’ needs, blended learning, and instructional design through the ADDIE model.

### 2.1. Population and sample group

The study included two groups of participants:

1. Approximately 1,000 undergraduate students were enrolled in basketball physical education courses at Wannan Medical College in Wuhu City, China. From this population, a sample of 286 students was selected using the Taro Yamane formula [28], and simple random sampling was employed to ensure equal selection probability for each individual.
2. A separate cohort of 30 undergraduate students from the Clinical Faculty participated in the development and evaluation of the training program, as well as the satisfaction survey. A power analysis using G*Power (v3.1.9.7) indicated that a minimum of 34 participants was required (effect size *d* = 0.5, *α *= 0.05, **power* *= 0.80). Although the final sample size was slightly below this threshold, it was considered acceptable in the context of class size limitations and the exploratory nature of the pilot study.

### 2.2. Research instruments and procedures

#### 2.2.1. Needs assessment questionnaire.

A 39-item questionnaire was adapted from previously validated blended learning surveys [29,30] to assess students’ needs related to blended basketball courses. Each item was rated on a five-point Likert scale (1 = Strongly disagree, 5 = Strongly agree).

A panel of five experts (three Thai and two Chinese) evaluated the content validity of the questionnaire using the Index of Item-Objective Congruence (IOC) [31]. Items with IOC values below 0.67 were revised or removed [32]. All retained items had an IOC of 1.00.

For reliability assessment, 30 students from Wannan Medical College completed a pilot version of the questionnaire. Cronbach’s alpha (*α*) was used to measure internal consistency [33], yielding a value of 0.99. On average, it took participants approximately 10–12 minutes to complete the questionnaire.

#### 2.2.2. Blended learning basketball training program.

To develop an administration model for the training program, the following steps were undertaken:

(1) A draft version of the training program was created for undergraduate students.
(2) Five experts evaluated the appropriateness and internal consistency of the program using a two-part training evaluation form. The first part which assessed appropriateness produced a mean score of 4.60 (SD = 0.53). The second part (consistency) was assessed via IOC, resulting in a value of 1.00.
(3) A pilot session with 30 clinical medicine students was conducted to refine the training content and procedures. Based on the feedback received, the finalized program was implemented over five training days distributed across a three-week period.

The training adopted a blended learning format. The online component, delivered via the Xuexi Tong platform, allowed instructors to upload instructional videos and PowerPoint slides, and to interact with students through a built-in messaging and discussion module. Students were able to review materials and submit questions at any time. The offline component consisted of in-person technical instruction, skill drills, and real-time feedback during class sessions.

All participants belonged to the same administrative class, which facilitated weekend scheduling and ensured organizational efficiency. Their existing familiarity with one another also supported effective peer interaction and group collaboration, key elements for skill-based physical education training conducted within a short period. A single evaluator assessed student performance using clearly defined rubrics to ensure scoring consistency.

A control group was not included due to both practical and pedagogical considerations. The fixed class schedule, limited training duration, and the likelihood of information exchange between groups made random assignment and between-group comparison impractical. Therefore, a one-group pre-test–post-test design was selected to allow concentrated implementation and formative evaluation of the training program.

#### 2.2.3. Satisfaction survey.

An undergraduate satisfaction survey was adapted from established blended levels of satisfaction scales [34] and was tailored to the basketball context. It contained six sections with a total of 30 questions rated on a five-point Likert scale. Content validity was confirmed by five experts (IOC = 0.98), and the questionnaire’s reliability was 0.96. Descriptive statistics (frequencies, percentages, means, standard deviations (SD)) were used to analyze the data.

To minimize response bias, several precautionary measures were implemented. Before completing the survey, student participants were informed that their responses would remain completely anonymous. They were also assured that their answers would not affect their course grades or performance evaluations. Additionally, students were explicitly encouraged to provide honest and candid feedback based on their actual learning experiences.

### 2.3. Ethical considerations

The study was approved by the Medical Ethics Committee of Wannan Medical College, China (Reference No. 2024-246), and by the Human Research Ethics Committee of Dhonburi Rajabhat University, Thailand (Reference No. DRUIRB-GOV-66-00015). All participants provided written informed consent prior to participation. Participant recruitment and training were conducted on weekends to avoid interfering with academic schedules.

### 2.4. Data analysis

Data from the needs assessment and satisfaction surveys were analyzed quantitatively. Descriptive statistics (frequencies, percentages, means, SD) were calculated to summarize participant characteristics and evaluate overall levels of need and satisfaction. Reliability was examined using Cronbach’s alpha (α), and content validity was determined via the IOC; items scoring below 0.67 were revised or removed in accordance with expert feedback. In the blended training program evaluation, expert ratings were aggregated to determine program appropriateness and consistency. For the training effectiveness analysis, a paired samples t-test was employed to compare students’ performance before and after the training. Shapiro–Wilk tests were conducted on the difference scores of each test item, and the results indicated that the normality assumption was generally satisfied, supporting the use of the paired samples t-test. Effect sizes were calculated using Cohen’s *d* (t/√n for paired samples) to evaluate the practical significance of the observed differences. All analyses were performed using SPSS version 27.0 and Microsoft Excel 2007.

## 3. Results

### 3.1. Results of student needs

The questionnaire designed to assess the needs of the blended basketball program included a sample of 286 students and was divided into two parts. The first part collects basic information about each participant, and the second part consists of seven categories with 39 questions designed to identify needs related to the learning environment of the blended basketball program. A five-point Likert scale is used for each item in the questionnaire and the assessment criteria are shown in Table 1.

**Table 1:** Likert scale evaluation criteria.

Rank	Current situation/ Expectation	Mean Range	Need Level
5	Highest	4.21–5.00	The most
4	High	3.21–4.20	A lot
3	Moderate	2.21–3.20	Moderate
2	Low	1.21–2.20	Little
1	Very Low	0.00–1.20	Minimal

According to Table 2, the study included a total sample of 286 university students, with 138 males (48.30%) and 148 females (51.70%), indicating a balanced gender distribution. The sample’s age is primarily concentrated in the 18–25 age range, with 170 students aged 18–20 (43.90%) and 116 students aged 21–25 (56.10%), while no participants are in the 26–30 age group. Overall, the sample consists entirely of university students, predominantly within the 18–25 age group.

**Table 2:** Student sample for the basketball blended learning training program.

General information		Total (n)=286	
		Frequency	Percentage
Gender	Male	138	48.30
	Female	148	51.70
Ages	18-20	170	43.90
	21-25	116	56.10
	26-30	0	0.00

**Fig 2:**
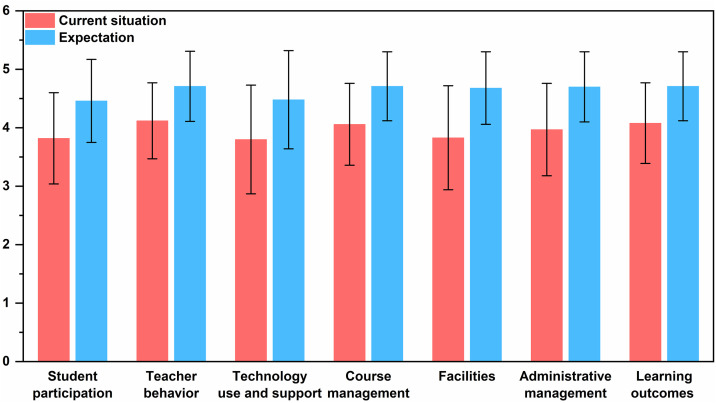
Students’ current situation and expectation regarding the blended learning environment.

According to Table 3 and Fig 2, which provides a comprehensive overview of the Student Needs Analysis for Blended Learning of Basketball Classes at Wannan Medical College, the results indicate high overall satisfaction with the current situation, rated at 3.96, which qualifies as the high standard. The expected rating for the future situation is even higher, at 4.63, classified as the highest standard.

**Table 3:** Results of student needs analysis for blended learning in basketball classes.

Questions	Current situation			Expectation		
	$\overset{―}{x}$	SD	Rating Scales	$\overset{―}{x}$	SD	Rating Scales
Student participation	3.82	0.78	High	4.46	0.71	Highest
Teacher behavior	4.12	0.65	High	4.71	0.60	Highest
Technology use and support	3.80	0.93	High	4.48	0.84	Highest
Course management	4.06	0.70	High	4.71	0.59	Highest
Facilities	3.83	0.89	High	4.68	0.62	Highest
Administrative management	3.97	0.79	High	4.70	0.60	Highest
Learning outcomes	4.08	0.69	High	4.71	0.59	Highest
**Total average**	**3.96**	**0.68**	**High**	**4.63**	**0.56**	**Highest**

The specific items are analyzed as follows:

Teacher Behavior: Students reported high satisfaction with how teachers manage interactions, with the current rating at 4.12 and the future expectation at 4.71, this reflects a desire for even more engaging and supportive teaching practices.

Learning Outcomes: The current learning outcomes are rated at 4.08, with an expected improvement to 4.71. This indicates that students hope to achieve enhanced academic performance and skill development through blended learning and expect higher overall learning outcomes.

Course Management: The management of the course currently holds a strong rating of 4.06, with students anticipating further improvements to a rating of 4.71. This expectation suggests a need for even more streamlined and responsive course administration.

### 3.2. Results of focus group meeting

As summarized in Table 4, the expert panel provided valuable feedback to refine the training program. Experts agreed that the course “comprehensively covers fundamental skills and tactics, making it suitable for beginners,” and emphasized that “the training modules provide a well-rounded approach to both individual techniques and team coordination.” In terms of blended instruction, one expert noted that “the combination of online video review and offline correction effectively supports students’ practical learning.” The experts also affirmed that the program was appropriately designed for first-year students, highlighting its “step-by-step structure and adaptability to students’ progress.” These expert insights confirmed the program’s alignment with instructional objectives and informed its final optimization.

**Table 4:** Results of Interviews with subject matter experts.

Theme	Description
1. Comprehensiveness of course content	The course comprehensively covers fundamental skills and tactics, suitable for beginners, providing a solid foundation for future learning.
	It includes dribbling, passing, shooting, three-step layup, and offensive/defensive coordination, offering students a well-rounded training in both individual skills and team tactics.
	The content is well-structured to ensure balanced development in both individual skills and team coordination.
2. Rational allocation of class Time	The course time allocation is reasonable, ensuring skill improvement and proper preparation for the exam.
	Core skills like dribbling, shooting, and the three-step layup take priority, ensuring sufficient practice for key skills.
	The exam project (three-step layup) receives adequate training time, ensuring optimal performance in the assessment.
3. Blended teaching design	Online learning through videos and PPT helps students preview content and review after class, reinforcing skills.
	Offline teaching focuses on practical exercises and real-time feedback, helping students apply what they have learned in real situations.
	The integration of online and offline learning caters to diverse learning needs, enhancing learning efficiency and practical performance.
4. Course difficulty and adaptability	The course is appropriately designed for freshmen, gradually helping them master fundamental skills.
	The step-by-step approach allows students to improve their technical level progressively.
	The course is flexible and adjusts according to students’ learning progress, ensuring that everyone can improve at their own pace.
5. Testing and evaluation methods	The use of itemized scoring provides a clear and accurate assessment of students’ mastery of skills.
	Each skill is scored across four levels, making it easy for instructors to assess students’ progress.
	The comprehensive evaluation covers all key skills, ensuring an overall assessment of students’ learning effectiveness.

**Fig 3:**
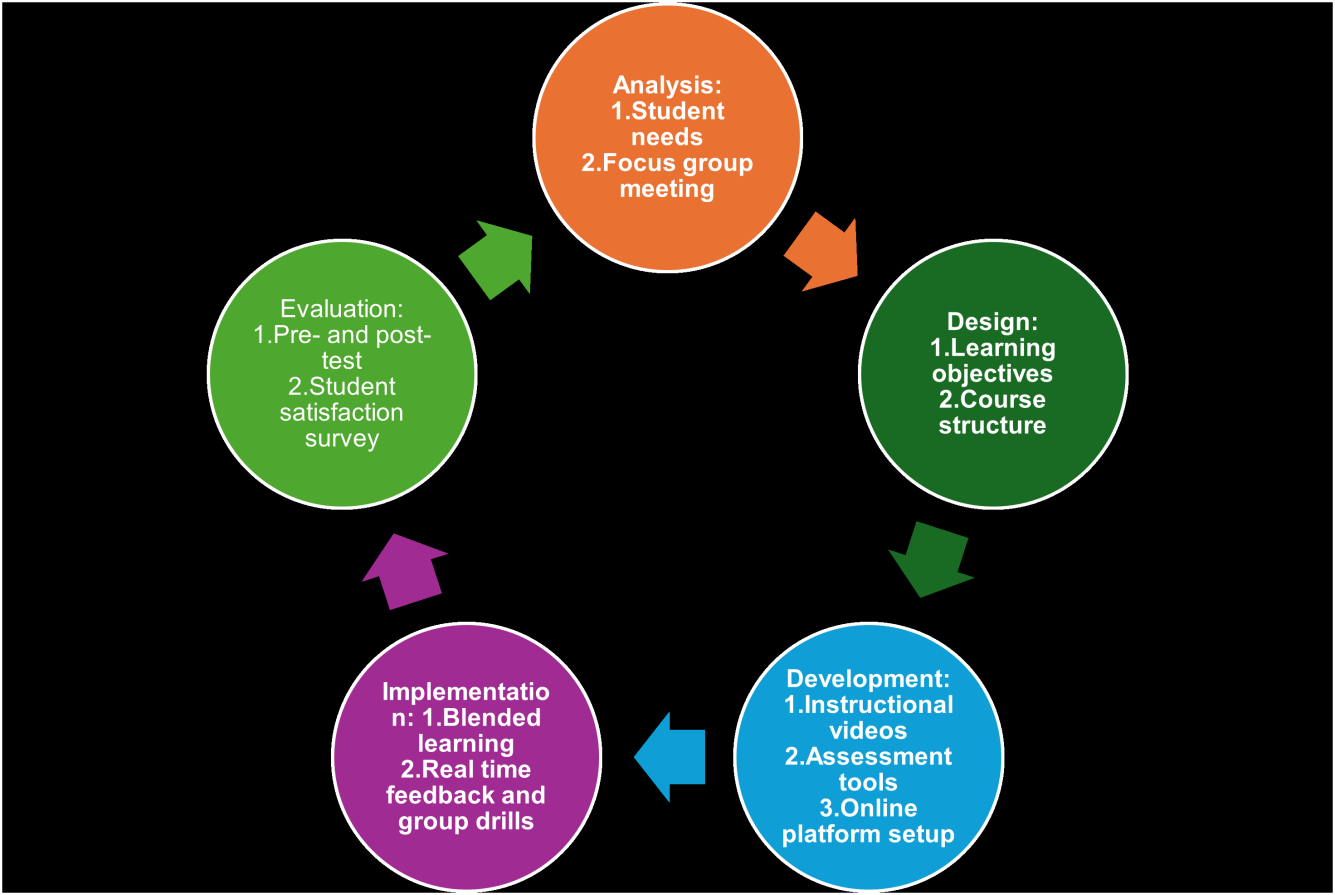
Implementation framework of the ADDIE model in the blended basketball training program.

The blended basketball training program model based on the ADDIE model designed in this study is shown in Fig 3 and Table 5. which detail how each stage of the ADDIE model was applied to guide the program’s design and implementation. The program spans 30 hours over 5 days, with 6 hours of training each day. The details are as follows:

**Table 5:** Summary of ADDIE model implementation in the blended basketball training program.

ADDIE Phase	Implementation Summary
Analysis	A needs assessment was conducted through student questionnaires and expert panel discussions to identify learning preferences and skill gaps. A baseline pre-test on fundamental basketball skills was also administered.
Design	Learning objectives were established based on the assessment results. Modular content was designed around core basketball skills, with clearly allocated training hours for each module. Both formative and summative assessments were planned, integrating online and offline instructional components.
Development	Instructional materials, including videos, PowerPoint slides, and standardized evaluation rubrics, were developed and uploaded to the Xuexi Tong platform to support self-directed learning and review. All skill assessments were conducted in person.
Implementation	Students accessed theoretical content and skill demonstrations online, while in-person sessions focused on technical practice, real-time corrective feedback, and peer collaboration. Group-based drills were emphasized to enhance both individual performance and teamwork.
Evaluation	Program effectiveness was evaluated using pre- and post-training skill tests and a satisfaction survey. Assessment covered dribbling, passing, shooting, three-step layups, and offensive–defensive coordination.

1. Analysis

A questionnaire was administered to assess students’ needs for the blended learning environment. A pre-test was also conducted to evaluate basic basketball skills, including dribbling, passing, shooting, and three-step layups. To ensure alignment between the training content and student needs, an expert panel discussion was held (see Table 4). Details of the expert information are summarized in Table 6.

**Table 6:** Basketball blended learning training program content.

Training Module	Training Content	Detailed Exercises	Training Goal
1. Dribbling	Basic dribbling skills	High dribbling; Low dribbling; Change-hand dribbling; Dribbling while moving	Improve ball control and dribbling agility
2. Passing	Various passing Techniques	chest pass; Ground pass; Passing while moving	Enhance passing accuracy and timing
3. Shooting	Shooting skills	Standing one-handed shoulder shot	Improve shooting stability and accuracy
4. Three-step layup	Layup technique	Three-step layup	Enhance driving ability and layup coordination
5. Offensive and defensive coordination	Offensive and defensive play coordination	Offensive coordination: pick-and-roll, cutting; Defensive coordination: man-to-man defense, zone defense, defensive footwork	Improve team coordination and defensive response speed

1. Design

Based on the results of the needs analysis, the program was designed with a focus on fundamental basketball skills. Each skill module was assigned specific training hours and learning objectives. A blended learning model was adopted, enabling students to acquire theoretical knowledge through online resources and to enhance practical abilities through offline physical training sessions.

3 Development

Instructional materials, including teaching videos and performance evaluation rubrics, were developed and uploaded to the online learning platform for students to access during self-directed learning. While assessments were conducted offline during classroom sessions, the online platform served as a supplementary tool for review and reinforcement.

4 Implementation

The program integrated online instruction with face-to-face training. Students engaged with video materials on the platform and participated in structured offline sessions focused on skill drills and instructor feedback. Repetitive practice and guided correction were emphasized to support skill mastery.

5 Evaluation

The effectiveness of the program was assessed through pre- and post-training tests covering dribbling, passing, shooting, and three-step layups. Additionally, a student satisfaction survey was administered to evaluate perceptions of the blended learning experience and instructional support. These resulting data provided evidence of the program’s effectiveness and offered insights for future refinement and enhancement.

### 3.3. Results of pre-test and post-test

To evaluate the effectiveness of the training program, a paired samples t-test was conducted to compare students’ performance before and after the intervention. Prior to the analysis, the Shapiro–Wilk test confirmed that the assumption of normality was generally satisfied, supporting the use of the paired t-test. The pre-test was administered before the training course and the post-test at the end of the course. The results of the statistical analysis are summarized in Table 6.

According to Table 7, all assessed basketball skills showed statistically significant improvement from the pre-test to the post-test after the implementation of the training program. Dribbling scores increased from a mean of 12.40 to 15.80 (*p* < .001, *d* = 2.14), and passing scores rose from 12.00 to 15.97 (*p* < .001, *d* = 3.34). Shooting performance improved from 10.17 to 13.10 (*p* < .001, *d* = 1.94), while scores for the three-step layup increased from 11.33 to 15.20 (*p* < .001, **d* *= 3.10). The most notable gains were observed in offensive and defensive skill sets, which improved from 10.03 to 15.20 (*p* < .001, *d* = 3.11). Additionally, the overall total score increased from 55.93 to 75.27 (*p* < .001, *d* = 3.65), indicating a substantial enhancement in students’ basketball performance following the intervention.

**Table 7:** Paired sample statistics (pre-test and post-test) blended learning.

Items	N	Pre-Test (Mean ± SD)	Post-Test (Mean ± SD)	95% CI of Difference	t	P	Cohen’s d
Dribbling	30	12.40** ± **2.98	15.80** ± **1.85	−3.99 to −2.81	−11.7	<.001	2.14
Passing	30	12.00 **± **2.42	15.97** ± **1.85	−4.41 to −3.52	−18.3	<.001	3.34
Shooting	30	10.17** ± **2.53	13.10** ± **2.54	−3.50 to −2.37	−10.7	<.001	1.94
Three-step layup	30	11.33** ± **2.68	15.20** ± **1.92	−4.33 to −3.40	−16.9	<.001	3.10
Offensive and defensive skill sets	30	10.03** ± **2.82	15.20** ± **1.92	−5.79 to −4.55	−17.0	<.001	3.11
**Total Score**	**30**	**55.93 ± 13.05**	**75.27 ± 9.06**	**−21.31 to −17.35**	**−20.0**	**<.001**	**3.65**

### 3.4. Results of student satisfaction

1. The analysis is conducted on 30 undergraduate students (aged 18–20 years, categorized by gender) from the Clinical Faculty of Wannan Medical College, majoring in Clinical Medicine. Among these participants, 56.7% (n = 17) are male and 43.3% (n = 13) are female. Data is analyzed using frequencies and means.
2. A survey of undergraduate students’ satisfaction with the training program. The results of the survey are shown in Table 7.

According to Table 8, the overall means of student satisfaction across all 30 items is 4.07 (SD = 0.86), which is recognized as a high level. Among the six items, overall satisfaction achieves the highest means of 4.36 (SD = 0.61), firmly at the highest level. Next is learning outcomes with 4.19 (SD = 0.84), also at a high level. This is followed by teaching quality at 4.03 (SD = 0.84) and learning resources at 4.02 (SD = 0.89), both classified as high levels. Course content then appears at 3.93 (SD = 0.92), likewise considered high, and the learning platform ranks last at 3.91 (SD = 0.95), yet still at a high level. These findings suggest that students particularly value tangible benefits (e.g., improved basketball skills), their overall experience, and the professional quality of both instruction and resources offered in the program.

**Table 8:** Results of students’ satisfaction with the blended learning basketball training program.

Items	Mean	SD	Rating Scales
Course content	3.93	0.92	High
Teaching quality	4.03	0.84	High
Learning resources	4.02	0.89	High
Learning platform	3.91	0.95	High
Learning outcomes	4.19	0.84	High
Overall satisfaction	4.36	0.61	Highest
**Average**	**4.07**	**0.86**	**High**

## 4. Discussion

This study aimed to develop and evaluate a basketball training program based on the ADDIE instructional design model. The study is conducted at a university in Anhui Province, China, where a five-day training program is implemented for 30 college students. The results indicate significant improvements in students’ basic skills, and the overall course satisfaction is high. Through questionnaire surveys and expert group discussions, we further validate the effectiveness and practicality of the course. The following discussion will focus on three key aspects: student needs, teaching effectiveness, and student satisfaction.

Firstly, regarding student needs, based on the survey results, we find that the top three areas of demand for the blended basketball course learning environment are teacher behavior, learning outcomes, and technical support. Students expressed high expectations for teacher support, better learning outcomes, and more stable technical platforms, highlighting the need to optimize instructional interaction, outcome effectiveness, and technical infrastructure [35,36]. Therefore, future course design should pay more attention to optimizing teacher behavior, enhancing the effectiveness of learning outcomes, and enhancing technical infrastructure to better meet students’ needs.

Secondly, this study comprehensively evaluated the instructional effectiveness across five core basketball skills: dribbling, passing, shooting, the three-step layup, and offensive-defensive coordination. The results revealed significant improvements in all skill areas, supporting the conclusion of Wang et al. [37] that blended learning can effectively enhance fundamental motor skills among university students. Among these skills, dribbling and passing demonstrated the most substantial gains, likely due to their lower motor complexity and the targeted instructional support provided. These skills involve fewer body segments and simpler coordination, making them more responsive to short-term, repetitive training. This aligns with motor skill complexity theory, which suggests that tasks with lower cognitive and biomechanical demands are more amenable to rapid improvement [38].

The three-step layup, a moderately complex integrative skill, also showed substantial improvement, indicating that the blended training program effectively supported learning progression for intermediate-level techniques. To further enhance learning outcomes, the use of instructional strategies such as tiered cooperative learning may help accommodate students with varying baseline abilities improve more efficiently [22]. In contrast, shooting exhibited the most modest improvement, likely due to its high-level coordination demands, including visual-motor alignment and timing precision [39]. These findings underscore the importance of differentiated instruction and extended training time for complex skills like shooting [40,41]. For offensive and defensive coordination, although the content was limited to basic tactical concepts, students exhibited significant gains, likely due to the very low starting point and structured instruction.

From a quantitative perspective, all five measured skills showed large to very large standardized effect sizes, with Cohen’s d ranging from 1.94 to 3.34. Specifically, passing and three-step layups exhibited the highest gains. A recent study reported a 28.0% improvement in total physical education skill scores through blended learning [42]. In our study, the total basketball skill score improved by 32.2%, further supporting the effectiveness of this instructional approach. Compared with previous studies, such as Wang et al. [23], which reported only moderate effect sizes (d = 0.46–0.56), the current program demonstrates substantially stronger instructional effects. These findings highlight the potential of well-structured blended training to enhance skill acquisition and support the use of differentiated, skill-specific strategies to meet learners’ diverse needs and optimize instructional impact.

Furthermore, the effectiveness of the blended training may be closely linked to its instructional structure. The self-paced online videos allowed students to repeatedly review key techniques, reinforcing motor memory and reducing cognitive load during offline sessions. Meanwhile, real-time instructor feedback during in-person practice enabled immediate correction and skill refinement, especially for complex movements like the three-step layup and shooting. This combination of independent review and guided feedback aligns with findings from Fleck et al. [43] and Hopla [39], who emphasize the complementary benefits of multimedia instruction and timely correction in motor skill acquisition. Students in this study appeared to benefit from both elements, improving basic and complex skills through targeted and structured blended instruction.

Finally, the results of the satisfaction survey indicate high overall student satisfaction with the course (mean score = 4.63 out of 5). This is comparable to findings from a recent study in which students participating in a blended learning environment reported a satisfaction score of 4.27 out of 5 [44]. Additionally, students rated both the course content and teaching quality highly, which is consistent with Fleck’s viewpoint. Both studies suggest that the combination of diverse online teaching resources and detailed skill explanations from instructors can effectively improve satisfaction [43]. However, there is still room for improvement in personalized guidance and the frequency of updating learning materials. Regarding the use of learning resources and platforms, although the overall satisfaction is high, student feedback indicates a need for improvements in search convenience and technical support [45]. In terms of learning outcomes, most students report significant improvements in their skill levels and expressed a desire for more training sessions that simulate real-game scenarios. Based on these findings, we believe that future improvements can be made in platform optimization, technical support, and the design of practical training sessions to better meet students’ diverse learning needs and enhance the overall instructional effectiveness of the course.

This study provides a theoretical foundation for integrating the ADDIE model into the design of physical education courses and holds significant practical value. The findings indicate that by combining the ADDIE model with student needs analysis, it is possible to effectively design and optimize basketball courses, enhance students’ foundational skills, and meet their diverse learning needs. The study’s strength lies in its systematic ADDIE-based design, refined through student needs analysis and expert input to ensure alignment with learners’ actual requirements.

However, this study has several limitations that should be acknowledged. First, the sample size was relatively small and limited to a single university, which may restrict the generalizability of the findings. Second, the short duration of the training program constrained the ability to evaluate long-term learning outcomes. Third, performance assessments were conducted by a single evaluator, which may introduce some degree of subjectivity even use standardized rubrics, Lastly, the use of one-group pre-test–post-test design without a control group may limit the strength of causal interpretations.

To address these limitations, future research could consider employing randomized controlled trials to enhance internal validity, including a larger and more diverse sample, and more evaluators engagement to improve scoring objectivity. In particular, longitudinal tracking of students’ skill retention and learning motivation over time would provide valuable insight into the long-term effectiveness of the program. In addition, it would be worthwhile to investigate gender-based differences in response to blended learning. Future studies could also explore the scalability of the ADDIE-based model to other sports, such as volleyball or soccer. These directions could help assess the model’s adaptability in different physical, instructional, and demographic settings.

## 5. Conclusion

This study demonstrated that a blended learning basketball training program developed using the ADDIE model effectively improved university students’ fundamental skills, such as dribbling and passing. Meanwhile, performance gains in more complex skills, including shooting and three-step layups, were comparatively modest, suggesting that additional instructional strategies may be needed in these areas. These findings support the feasibility of applying the ADDIE model in physical education settings and contribute to the growing evidence on blended learning for skill-based sports instruction. The results indicate that structured, blended approaches can help address student learning needs in a practical and flexible manner. Future research is recommended to examine long-term skill retention, implement randomized controlled trials, and explore the model’s adaptability across different sports and institutional contexts to further validate and expand upon these findings.

## Supporting information

S1 Appendix Questionnaire 1. (DOCX)

S2 Appendix Questionnaire 2. (DOCX)

S3 Appendix Focus group meeting result. (DOCX)

S4 Appendix Pre- and post-test scores. (XLSX)
