# Supplementary material for: Design and evaluation of a blended basketball training program using the ADDIE model
Source: PLoS One. 2025 Sep 29;20(9):e0332820. doi: 10.1371/journal.pone.0332820 (PMC12478884; doi:10.1371/journal.pone.0332820)
Supplement: S1 Appendix — (DOCX) [file pone.0332820.s001.docx]

**Appendix A: The Questionnaire of Students Needs Assessment**

**Instruction: There are 2 sections:**

Section 1: General Information

Section 2: The learning environment needs assessment for an administration model of a training program by using blended Learning for Basketball Classes at Wannan Medical College, Wuhu, China.

**Section 1: General Information**

Please check (√) in the blank given:

1) Gender:

___Male ___Female

2) Years of Age :

___18-20 age ___21-23 age

**Section 2:** The Learning environment needs of an administration model of a training program by using blended Learning for Basketball Classes at Wannan Medical College, Wuhu, China.

| **Questions** | **What is?** | | | | | **What Should be?** | | | | |
| --- | --- | --- | --- | --- | --- | --- | --- | --- | --- | --- |
|  | Lowest  （1） | Low  （2） | Middle  （3） | High  （4） | Highest  （5） | Lowest  （1） | Low  （2） | Middle  （3） | High  （4） | Highest  （5） |
| **Student Participation** | | | | | | | | | | |
| 1. The level of interaction with peers during online and in-person sessions. |  |  |  |  |  |  |  |  |  |  |
| 2. Opportunities for group work and collaborative projects in the course. |  |  |  |  |  |  |  |  |  |  |
| 3. The effectiveness of discussion forums or other interactive platforms in facilitating class engagement. |  |  |  |  |  |  |  |  |  |  |
| 4. Your ability to engage in meaningful discussions with the instructor and classmates. |  |  |  |  |  |  |  |  |  |  |
| 5. The frequency and quality of feedback received from peers and instructors. |  |  |  |  |  |  |  |  |  |  |
| 6. The encouragement from instructors to participate actively in learning activities. |  |  |  |  |  |  |  |  |  |  |
| **Teacher behavior** | | | | | | | | | | |
| 7. The teacher's leadership style has a positive impact on your learning attitude and motivation. |  |  |  |  |  |  |  |  |  |  |
| 8. The teacher effectively interacts with students to enhance their learning experience. |  |  |  |  |  |  |  |  |  |  |
| 9. The teacher provides guidance that helps you understand and master basketball skills. |  |  |  |  |  |  |  |  |  |  |
| 10. The teacher offers timely feedback that aids in improving your learning methods and skills. |  |  |  |  |  |  |  |  |  |  |
| 11. The teacher encourages you, boosting your confidence and motivation. |  |  |  |  |  |  |  |  |  |  |
| 12. The teacher demonstrates adequate professional knowledge in teaching basketball. |  |  |  |  |  |  |  |  |  |  |
| 13. The teacher treats all students fairly, regardless of their skill levels. |  |  |  |  |  |  |  |  |  |  |
| 14. The teacher seriously considers students' opinions and questions, providing appropriate responses. |  |  |  |  |  |  |  |  |  |  |
| **Course Management** | | | | | | | | | | |
| 15. The course schedule is well-organized and communicated in advance. |  |  |  |  |  |  |  |  |  |  |
| 16. The course objectives are clear and aligned with the training activities. |  |  |  |  |  |  |  |  |  |  |
| 17. Course materials and resources are readily available and useful. |  |  |  |  |  |  |  |  |  |  |
| 18. The technology used in the course enhances learning. |  |  |  |  |  |  |  |  |  |  |
| 19. Feedback from students is regularly solicited and used to improve the course. |  |  |  |  |  |  |  |  |  |  |
| 20. The course offers flexibility to accommodate students’ various learning needs. |  |  |  |  |  |  |  |  |  |  |
| 21. The assessment methods are transparent and contribute to fair grading. |  |  |  |  |  |  |  |  |  |  |
| **Technology use and support aspects** | | | | | | | | | | |
| 22. The online learning platform is easy to use. |  |  |  |  |  |  |  |  |  |  |
| 23. Help is easily accessible when I encounter difficulties with the platform. |  |  |  |  |  |  |  |  |  |  |
| 24. Technical issues frequently interfere with my learning. |  |  |  |  |  |  |  |  |  |  |
| 25. The technology used in the course enhances my overall learning experience. |  |  |  |  |  |  |  |  |  |  |
| **Facility** | | | | | | | | | | |
| 26. The basketball courts are regularly maintained and suitable for training sessions. |  |  |  |  |  |  |  |  |  |  |
| 27. During practice, there is enough equipment like basketballs and practice markers available for all students. |  |  |  |  |  |  |  |  |  |  |
| 28. The indoor gym is well ventilated and has adequate temperature control for comfortable training sessions. |  |  |  |  |  |  |  |  |  |  |
| 29. The lighting in the training areas is always sufficient for safe and effective practice. |  |  |  |  |  |  |  |  |  |  |
| 30. Safety measures and emergency equipment like first aid kits are easily accessible during training sessions. |  |  |  |  |  |  |  |  |  |  |
| 31. The overall cleanliness and upkeep of the training facility meet high standards. |  |  |  |  |  |  |  |  |  |  |
| **Administration** | | | | | | | | | | |
| 32. Administrative support is readily available and helpful when needed. |  |  |  |  |  |  |  |  |  |  |
| 33. The administrative procedures for handling student inquiries and issues are efficient. |  |  |  |  |  |  |  |  |  |  |
| 34. The institution's communication channels (e.g., email, portals) are reliable and provide timely updates. |  |  |  |  |  |  |  |  |  |  |
| **Learning outcomes and satisfaction** | | | | | | | | | | |
| 35. The basketball skills taught in this program align closely with my personal interests. |  |  |  |  |  |  |  |  |  |  |
| 36. The skills I have learned will improve my performance. |  |  |  |  |  |  |  |  |  |  |
| 37. I am satisfied with the teaching methods. |  |  |  |  |  |  |  |  |  |  |
| 38. This training has met my expectations. |  |  |  |  |  |  |  |  |  |  |
| 39. I would recommend this training program to others. |  |  |  |  |  |  |  |  |  |  |
