# Supplementary material for: Design and evaluation of a blended basketball training program using the ADDIE model
Source: PLoS One. 2025 Sep 29;20(9):e0332820. doi: 10.1371/journal.pone.0332820 (PMC12478884; doi:10.1371/journal.pone.0332820)
Supplement: S2 Appendix — (DOCX) [file pone.0332820.s002.docx]

**Appendix B: The Questionnaire for Students Satisfaction**

**Instruction: There are 2 sections:**

Section 1: General Information

Section 2: The Satisfaction for an administration model of a training program by using blended Learning for Basketball Classes at Wannan Medical College, Wuhu, China.

**Section 1: General Information**

Please check (√) in the blank given:

1) Gender:

___Male ___Female

2) Years of Age:

___18-20 age ___21-23 age

**Section 2:** The Satisfaction for an administration model of a training program by using blended Learning for Basketball Classes at Wannan Medical College, Wuhu, China.

| **Questions** | **Level of Satisfaction** | | | | | **Detailed description** |
| --- | --- | --- | --- | --- | --- | --- |
|  | Highest  -5 | High  -4 | Middle  -3 | Low  -2 | Lowest  -1 |  |
| **Course Content** | | | | | | |
| 1. I am satisfied with the theoretical knowledge provided in the course. |  |  |  |  |  |  |
| 2. I am satisfied with the practical skills training provided in the course. |  |  |  |  |  |  |
| 3. I find the course content to be rich, interesting, and engaging. |  |  |  |  |  |  |
| 4. I believe the course covers all necessary basketball skills and knowledge. |  |  |  |  |  |  |
| 5. I am satisfied with the frequency of updates and improvements to the course content. |  |  |  |  |  |  |
| **Teaching Quality** | | | | | | |
| 6. I am satisfied with the professionalism and teaching abilities of the instructors. |  |  |  |  |  |  |
| 7. I am satisfied with the frequency and quality of interactions between instructors and students in the class. |  |  |  |  |  |  |
| 8. I am satisfied with the instructors' classroom administration and organizational skills. |  |  |  |  |  |  |
| 9. I feel that instructors adequately address my individual learning needs. |  |  |  |  |  |  |
| 10. I am satisfied with the timeliness and effectiveness of the feedback provided by instructors. |  |  |  |  |  |  |
| **Learning Resources** | | | | | | |
| 11. I am satisfied with the availability and quality of instructional videos for basketball learning. |  |  |  |  |  |  |
| 12. I find interactive learning platforms effective for communicating with other learners and instructors. |  |  |  |  |  |  |
| 13. I value the personalized feedback from instructors on my basketball skills development. |  |  |  |  |  |  |
| 14. I am satisfied with the technical support team's responsiveness to issues with online learning resources. |  |  |  |  |  |  |
| 15. I believe access to an online resource library and reference materials is crucial for improving learning efficiency. |  |  |  |  |  |  |
| **Learning Platform and Technical Support** | | | | | | |
| 16. I am satisfied with the user-friendliness and ease of use of the learning platform. |  |  |  |  |  |  |
| 17. I appreciate the variety of interactive tools, such as forums and discussion groups, provided by the platform. |  |  |  |  |  |  |
| 18. I am satisfied with the speed and effectiveness of the technical support when encountering technical issues. |  |  |  |  |  |  |
| 19. I find the learning platform to be stable and reliable for my study needs. |  |  |  |  |  |  |
| 20. The platform performs well and is compatible across different devices, which meets my learning needs. |  |  |  |  |  |  |
| **Learning Outcomes** | | | | | | |
| 21. I am satisfied with my mastery of basketball theoretical knowledge after completing the course. |  |  |  |  |  |  |
| 22. I am satisfied with the improvement of my basketball practical skills after the course. |  |  |  |  |  |  |
| 23. I feel that the course has significantly contributed to my overall progress in basketball learning. |  |  |  |  |  |  |
| 24. The course has effectively enhanced my understanding and application of basketball tactics and strategies. |  |  |  |  |  |  |
| 25. I am satisfied with the performance improvement in basketball games because of this course. |  |  |  |  |  |  |
| **Overall Satisfaction** | | | | | | |
| 26. Overall, I am very satisfied with the blended basketball training program. |  |  |  |  |  |  |
| 27. The course met my expectations in terms of learning support and resources. |  |  |  |  |  |  |
| 28. The program has significantly supported my personal development goals in basketball. |  |  |  |  |  |  |
| 29. I would recommend this blended basketball training program to others interested in improving their skills. |  |  |  |  |  |  |
| 30. I am satisfied with my overall experience participating in this basketball training program and believe it was worth my time and effort. |  |  |  |  |  |  |
