# Supplementary material for: Design and evaluation of a blended basketball training program using the ADDIE model
Source: PLoS One. 2025 Sep 29;20(9):e0332820. doi: 10.1371/journal.pone.0332820 (PMC12478884; doi:10.1371/journal.pone.0332820)
Supplement: S3 Appendix — (DOCX) [file pone.0332820.s003.docx]

**Focus group meeting result**

To ensure the basketball training program aligns with university students’ learning needs and maintains scientific rigor in its content, methodology, and evaluation, a focus group meeting was conducted. The meeting brought together 10 experts from the fields of basketball education, sports training, and curriculum design to thoroughly discuss and evaluate the program’s structure and implementation.

The discussion focused on five key aspects: comprehensiveness of the course content, rationality of time allocation, blended teaching design, adaptability to freshmen’s needs, and testing and evaluation methods. During the meeting, the experts provided specific recommendations regarding the coverage of basketball skills, the appropriateness of teaching hours, the effectiveness of combining online and offline teaching, the suitability of course difficulty for freshmen, and the scientific validity of the assessment methods.

This section presents the main outcomes of the focus group meeting, focusing on expert suggestions for optimizing course content, ensuring the rationality of the instructional design, and improving evaluation methods to support the refinement and enhancement of the training program.

**1. Comprehensiveness of the Course Content**

The expert panel unanimously agreed that the course content comprehensively covers the key foundational basketball skills and basic tactics, which are well-suited to the needs of beginners and lay a solid foundation for future learning. The core content includes dribbling, passing, shooting, three-step layup, and offensive/defensive coordination, providing students with a comprehensive training program from individual technical skills to team tactics.

Dribbling: The course includes training in both high dribbling and low dribbling, ensuring that students can flexibly apply dribbling techniques in different game tempos. Experts pointed out that dribbling is a core fundamental skill in basketball and mastering it will help students control the ball and move smoothly on the court.

Passing: Through exercises in chest passes and bounce passes, the course helps students improve their passing accuracy and consistency. Experts emphasized that passing is the foundation of team coordination, and the efficiency and precision of passes during offensive plays directly affect game outcomes. Thus, passing training is crucial.

Shooting: The course pays particular attention to one-handed shoulder shooting and the three-step layup, both of which are essential scoring techniques. Experts believe that mastering these techniques not only improves students' scoring ability but also helps them adapt quickly to various offensive strategies in games.

Three-step layup: As a core scoring skill, the three-step layup integrates dribbling, footwork coordination, and shooting. Experts unanimously agreed that training in the three-step layup is vital for improving students' offensive capabilities. Through continuous practice, students will be able to better control their rhythm and improve body coordination, thereby increasing their scoring efficiency in real-game situations.

Offensive and defensive coordination: The course also includes basic training in offensive and defensive tactics, with a focus on pick-and-roll plays and man-to-man defense. Experts highlighted that these tactics are crucial in team basketball, as they help students understand how to effectively collaborate within a team and apply tactics flexibly during matches.

The expert panel concluded that the course content is comprehensive and provides students with a solid basketball foundation. Overall, the course not only covers the basic technical skills of basketball but also introduces fundamental tactics, allowing students to achieve a balanced development of both individual skills and team coordination.

**2. Allocation of class time**

In the expert panel meeting, there was a discussion regarding the adjustment of course hours, particularly focusing on the half-court three-step layup as the key exam project. After careful deliberation, the expert panel unanimously agreed that the adjusted hour allocation is more reasonable, effectively supporting skill improvement and preparing students adequately for the exam. The specific hour allocation is as follows: Dribbling (6 hours), Shooting (6 hours), Three-Step Layup (9 hours), Passing (3 hours), Offensive Coordination (3 hours), Defensive Coordination (3 hours).

Dribbling (6 hours): As the core foundational skill in basketball, 6 hours of practice is deemed appropriate. Dribbling is involved in nearly every aspect of the game, especially during half-court transitions between offense and defense. Mastery of dribbling can significantly improve a player's control over the game tempo. Therefore, 6 hours of training ensures that students can master basic techniques like high and low dribbling and become proficient at ball control while moving.

Passing (3 hours): The expert panel unanimously agreed that 3 hours for passing training is suitable. Passing is a fundamental skill in team basketball, and although it is very important, the priority of passing exercises is lower compared to the exam projects. Three hours is sufficient for students to master basic passing techniques such as chest passes and bounce passes, meeting the needs for coordination at the beginner level.

Shooting (6 hours): Shooting is a key scoring skill in basketball, and the allocation of 6 hours ensures that students have enough time to practice basic shooting techniques. For beginners, mastering basic techniques such as the one-handed shoulder shooting is essential. Six hours of practice is deemed appropriate, as it will help improve the accuracy and consistency of their shooting.

Three-Step Layup (9 hours): Given that the three-step layup is the exam project for this course, the allocation of 9 hours is very reasonable. This skill involves dribbling, footwork coordination, and rhythm control during offense, requiring a significant amount of practice time. Experts believe that 9 hours of practice is sufficient for students to consolidate the skill from basic to game-specific applications, ensuring better performance during the assessment.

Offensive Coordination (3 hours): The training in offensive coordination focuses on enhancing students' ability to execute basic team tactics, such as pick-and-roll and cuts. While 3 hours of training might seem limited, it is deemed adequate for beginners to learn the fundamentals of offensive teamwork. Experts suggested that incorporating practice in game simulations could allow students to apply offensive tactics in real-game scenarios.

Defensive Coordination (3 hours): Defensive coordination is equally important in team basketball. However, considering the overall course duration, 3 hours allocated for defensive tactics is seen as sufficient for beginners. These hours focus on basic defensive techniques such as man-to-man defense, helping students understand team defense principles and how to execute them in practice.

**3. Blended Teaching Design**

The expert panel highly recognized the blended teaching design of this course, especially the combination of online and offline teaching methods, which maximize the advantages of both approaches.

Online Learning: The experts unanimously agreed that utilizing the "Rain Classroom" platform for online learning greatly benefits the students. Through resources like short videos and teaching PowerPoint slides, students can independently preview essential skills such as dribbling, passing, and shooting before the class. This model allows students to familiarize themselves with the teaching content in advance, improving the efficiency of offline practice. Additionally, the video resources also serve as review materials for students after class, reinforcing the technical points covered during lessons and helping students consolidate their learning and practice, especially for techniques prone to errors. Online resources play an important role in correcting such mistakes.

Offline Teaching: In the offline portion of the course, the focus is on practical exercises and real-time feedback from instructors. Experts emphasized that basketball, being a skill-based course, requires students to continuously practice and adjust their techniques in real-life scenarios. The instructor’s immediate feedback on the sidelines helps students promptly correct technical errors, particularly during training in dribbling, shooting, and three-step layup—core skills in basketball. Offline teaching enables students to integrate theoretical knowledge learned online with hands-on practice, allowing them to understand and apply the skills in real-game situations.

Advantages of Blended Teaching: The experts pointed out that this blended approach, combining online and offline learning, is highly effective for skill-based courses. The online component provides flexible learning arrangements, catering to students with different learning paces, while the offline part enhances learning outcomes through practical application and feedback. This combination ensures that students can independently master theoretical knowledge through online learning and simultaneously gain practical experience in real-life situations under the guidance of instructors.

Enhancing Interactivity: Experts suggested adding more interactive elements to the online portion, such as regular online discussions, Q&A sessions, or quizzes. These activities can help students better understand the key techniques in the videos and further stimulate their learning interest. This would improve the effectiveness of online learning and better prepare them for the offline practice sessions.

The expert panel unanimously agreed that the blended teaching design of the course is highly rational and effective. The integration of online and offline learning not only enhances students' ability to learn independently but also reinforces their technical proficiency through practical exercises and real-time feedback. The overall design helps improve students’ mastery of basketball skills and lays a solid foundation for their performance in actual games.

**4. The Needs of Student**

When discussing whether the course meets the learning needs of student, the expert panel unanimously agreed that the course’s difficulty level is appropriately designed, especially for freshmen to gradually learn and master fundamental basketball skills.

Reasonable Skill Difficulty, Experts pointed out that the course’s three-step layup, one-handed shoulder shooting, and basic defensive techniques are simple and easy to understand, making them very suitable for beginners. Through repeated practice of these foundational skills, students can steadily improve their basketball abilities without feeling overwhelmed by overly complex techniques.

Progressive Skill Improvement, the course adopts a step-by-step design, ensuring that students can gradually enhance their coordination and overall skills after mastering the basic techniques. Experts particularly appreciated this aspect, believing that beginners need to start with simple skills and gradually improve through consistent practice, boosting their confidence and technical proficiency along the way.

Flexibility for Adjustments, Experts also recommended that the course should maintain a certain level of flexibility to accommodate the varying learning speeds of different students. For instance, students who progress more quickly can be challenged with more advanced techniques, while those who are slower can benefit from additional basic exercises to consolidate their core skills.

Overall, the course design is well-structured, with an appropriate level of difficulty, helping students master basic basketball techniques and tactics within a limited timeframe. At the same time, the course’s flexibility ensures that every student can experience success and progress according to their individual learning pace. The section on "Comprehensiveness of Course Content" focuses on listing specific skills and their applications in basketball, while the section on "Meeting the Needs of Student" emphasizes the course's skill level and progressive nature, ensuring that students can learn in a step-by-step manner. This structure avoids repetition while highlighting the distinct focus of the course design.

**5. Testing and Evaluation Methods**

The expert panel unanimously agreed that the testing and evaluation methods of the course are both scientific and reasonable. The course assessments cover the key skills of basketball training, and through itemized scoring, the students’ technical mastery and progress can be comprehensively evaluated. The scores are divided into five categories, each worth 20 points, with a final total score of 100. The scoring is divided into four ranges: 16-20 points, 11-15 points, 6-10 points, and 1-5 points, which avoids overly fine or vague distinctions and makes the evaluation more intuitive and practical.

Specific Evaluation Content and Criteria:

Dribbling (20 points)

Test Content: 20-meter shuttle dribbling test to assess the student's ball-handling ability and movement speed on the court.

Scoring Criteria:

16-20 points: Fast speed with stable ball control, smooth movements with no errors.

11-15 points: Moderate speed with generally stable ball control, minor mistakes.

6-10 points: Average speed with unstable ball control, noticeable mistakes.

1-5 points: Slow speed with unstable ball control, frequent mistakes.

Passing (20 points)

Test Content: Chest pass and ground pass accuracy test to assess students' passing precision and control.

Scoring Criteria:

16-20 points: Precise passes with high stability, accurately reaching the target.

11-15 points: Mostly accurate passes with occasional deviation.

6-10 points: Average stability, noticeable deviation in passes.

1-5 points: Multiple inaccurate passes with clear deviations.

Shooting (20 points)

Test Content: 10 attempts of one-handed shoulder shooting, with the number of successful shots recorded to assess the students' shooting accuracy and consistency.

Scoring Criteria:

16-20 points: 8 or more successful shots, stable shooting technique.

11-15 points: 6-7 successful shots, shooting is generally on target.

6-10 points: 4-5 successful shots, shooting technique lacks stability.

1-5 points: 2-3 successful shots, noticeable errors in shooting form.

Three-Step Layup (20 points)

Test Content: Dribble and complete a three-step layup on half-court to assess footwork coordination and layup technique.

Scoring Criteria:

16-20 points: Smooth, standardized movement with fast speed and successful layup.

11-15 points: Basic standardized movement with moderate speed and minor errors.

6-10 points: Uncoordinated movement, noticeable mistakes, slow speed.

1-5 points: Poorly executed movement with multiple mistakes and slow speed.

Offensive and Defensive Coordination (20 points)

Test Content: Team coordination exercises or simulated games to assess students’ teamwork and tactical execution in offense and defense.

Scoring Criteria:

16-20 points: Seamless teamwork, full execution of tactics.

11-15 points: Tactics are mostly executed correctly, with occasional errors.

6-10 points: Tactical coordination lacks smoothness, with frequent errors.

1-5 points: Lack of coordination, insufficient execution of tactics.

6. Summary and Recommendations

The expert panel unanimously agreed that this course provides comprehensive and systematic basketball training for college student. The course content covers key skills such as dribbling, passing, shooting, three-step layup, and offensive and defensive coordination, fully meeting the training needs of beginners. The time allocation is reasonable, with a clear focus on key skills, ensuring that students can master and reinforce essential skills within a short period. The course uses scientific testing and evaluation methods with clear itemized scoring, making it easy to assess students' progress accurately and objectively.

The blended teaching design of the course, combining online previews and offline practical sessions, fully leverages the advantages of both self-directed learning and practical guidance. Students can review teaching videos and key points on the "Rain Classroom" platform before class and then apply what they have learned during offline practice sessions with real-time feedback from the teacher. This teaching model offers flexible learning arrangements, effectively improving both learning efficiency and practical performance.

Overall, the course design is scientific and well-reasoned, with highly practical evaluation methods that effectively improve students' foundational basketball skills, providing a solid foundation for their future basketball learning and team sports participation. By incorporating more online interaction, enhancing tactical exercises, and continuously optimizing evaluation standards, the course will become even more comprehensive, offering students a richer learning experience and greater opportunities for growth.
